# Supplementary material for: Are carbon emissions trading and green financial instruments synergistic? -Comprehensive quantitative research based on content analysis
Source: PLoS One. 2024 Mar 7;19(3):e0298601. doi: 10.1371/journal.pone.0298601 (PMC10919601; doi:10.1371/journal.pone.0298601)
Supplement: S1 Data — (ZIP) [file pone.0298601.s001.zip › Data/地级市Z_F工作报告.zip!/─┌├╔╣┼/░n═╖╩╨/└┤╫╘╣1⁄2╓┌║┼í╛┬φ┐╦╩2╛▌═°í┐_2014.docx]

各位代表：

现在，我代表县人民政府向大会报告工作，请予审议，并请县政协委员和列席会议的同志们提出意见。

一、2013年工作回顾

2013年，在市委、市政府和县委的正确领导下，在省级领导对口联系帮扶贫困县的大力推动下，在县人大、政协的监督和支持下，在全市各地区、部门、单位及社会各界的关心与帮助下，县政府团结带领全县各族人民，以科学发展观为指导，坚定不移地向脱贫致富奋力迈进，圆满完成了十六届人大一次会议确定的各项目标任务。

——主要经济指标提质增长。2013年，地区生产总值完成112.21亿元，较上年增长9.5%；固定资产投资完成141.42亿元，较上年增长18.2%；社会消费品零售总额完成16.56亿元，较上年增长11.1%；将地方财政总收入目标由11.5亿元调减为6.71亿元，实际完成7.15亿元，增长6.6%，进一步做实了财政，为下一步争取上级更大的支持奠定了基础。三次产业比重为12.4:70:17.6。编制完成《固阳县县域经济发展规划（2013—2017年）》，非公经济占经济总量的比重达到90%以上。

——工业经济平稳运行。面对严峻复杂的宏观经济形势，坚持稳工业、扶企业，与企业共度难关，实现政府、企业、就业三赢。为30家企业争取自治区电力扶持政策资金、市政府电价补贴、铁精粉临时补贴、中小企业贷款贴息共计2500万元，县财政配套1300万元。以园区为载体的工业主导产业继续巩固提升。园区一期基础设施累计完成投资1.7亿元，建设道路7.8公里、管网56.5公里；铁路专用线、管道天然气项目即将开工。实施工业重点项目17个，累计完成投资33.1亿元。包钢240万吨球团项目实现达产达效，汇豪10万吨镁合金综合技改5000吨棒材项目点火试炉，立富工贸煤化工120万吨洗精煤项目完成基础工程，科韵环保矿渣微晶玻璃材料项目正在建设厂房。与稀土高新区正式开展合作共建园区，与内蒙古科技大学在园区联合建设矿冶废弃物资源综合利用工程研究中心，成功引进了总投资4亿元的安徽和电普华高性能稀土铝合金电缆生产项目。

——农牧业产业健康发展。设施农业继续扩面增量，新增和改善节水灌溉面积4.1万亩，全县滴灌面积达到16万亩，覆膜、高垄栽培等抗旱技术得到进一步推广应用。马铃薯种植面积继续保持稳定，微型薯生产能力达到4000万粒，脱毒种薯应用10万亩，优质种薯在山东、广西、云南等地试种。农村合作组织达到368家，流转土地累计达到83万亩。羊存栏量和出栏率稳步提高，燕麦、黄芪和野猪、土鸡等特色种养业起步发展。田丰、草原百盈、三主粮等农牧业产业龙头企业成为引领农牧业产业化发展、带动农民增收的主导力量。

——第三产业稳步推进。传统服务业得到巩固，服务水平不断提高。重新修编了全县旅游发展规划，马鞍山景区道路建成通车，春坤山景区遗留问题得到解决，已签订开发协议，大仙山景区招商工作全面展开，投资70万元建成秦长城历史文化主题馆。田丰仓储能力达到6万吨，投资500万元的鸿盛达物流园区等物流项目已完成前期筹备工作。广众国际中心、城市广场和美源豪庭即将投入使用。

——城乡建设统筹发展。修编完成城市总规、外五镇总规及91个村庄建设规划。总投资4.5亿元完成城区道路、地下管网、供热管网、换热站等17项重点城建项目。投资1200万元完成3片区8万平方米的小街巷治理。历时三年多的城市棚户区改造取得决定性突破，回迁安置工作稳步推进。完成农村危房改造550户，总投资283万元新建农村饮水安全工程16处，解决了2700多人的安全饮水问题。完成2000座农村卫生改厕任务。实施了总投资4亿元220公里的省县乡公路路网建设工程。总投资27.2亿元的固阳至武川一级公路签订投资开发框架协议。完成总投资2500万元的电网改造工程。

——社会事业深入推进。投资2500万元完善学校基础设施建设，建成少年宫1座，迁建民族幼儿园完成主体建设，新建光明幼儿园开工建设。全县94%的贫困生得到救助，投入100万元奖励做出突出贡献的一线教师。投资120万元继续实施贫困寄宿生免费就餐项目。深化医药卫生体制改革，基本药物制度和零差价销售覆盖镇村两级医疗机构，基本建成县镇村三级公共卫生服务体系。开工建设县医院综合楼和兴顺西镇卫生院，新建25个村卫生室。为全县1.2万名65岁以上老人进行免费体检。成功举办了第十届“秦长城文化旅游节”，总投资9000万元、建筑面积1.78万平方米的县文化艺术中心已完成基础工程，建成4个镇小型健身广场。编制完成怀朔镇古城遗址保护方案，投资500万元建成长城保护工作站。完成2.7万套广播电视“户户通”工程，实现全覆盖。完成总投资600万元的山洪灾害防治非工程措施项目。二轮县志修编工作全面启动。

——民生保障持续改善。在财政收支矛盾日益突出的情况下，坚持优先保障民生投入，民生支出占财政支出的54.7%。城乡居民收入分别达到22394元和9155元，分别较上年增长9.2%和12.5%。为群众兴办的10件好事实事全部落地。发放创业贷款3800万元，全年城镇新增就业1500人，城镇登记失业率控制在3.8%。城乡低保实现与市区同步提高，城市低保标准提高到每人每月500元，农村低保标准提高到每人每年4756元，从三季度开始，全县75岁以上在村居住的非工资性收入且子女无赡养能力的贫困老人全部享受农村低保，从四季度开始，全县65岁以上在村居住的非工资性收入且子女无赡养能力的贫困老人逐步享受农村低保，全年发放低保、五保、医疗等救助金8000万元。城镇职工基本养老保险参保人数达到2.25万人，城乡居民养老保险参保人数达到10.3万人。农村新型合作医疗参合率达到99.2%，较去年提高0.14个百分点。全面贯彻自治区惠民政策，积极开展“一吨煤”送温暖活动。

——扶贫攻坚强力推进。《固阳县扶贫开发规划（2013—2017年）》获得自治区政府批复，11.8亿元的扶贫项目资金列入中央和自治区盘子。“全市集中社会力量重点帮扶固阳县”工作和市、县干部“一对一”帮扶活动深入开展，到位帮扶资金物资共计4387万元。完成了全县贫困人口的摸底调查工作，实现了贫困户信息化管理。

——生态环境治理全面启动。组织实施了三北防护林和碳汇造林等林业重点工程10.8万亩，投入1300万元完成一级公路通道绿化824亩、镇村道路绿化10公里。全面启动造林绿化和环境治理工作，制定了《固阳县造林绿化总体规划（2014—2018年）》，计划利用五年时间，总体投入绿化资金10.4亿元，绿化总面积7.5万亩。大力实施污染物减排工程，完成4家冶炼企业的减排工作，关停违法排污企业8家。“打非治违”和打击私挖盗采取得重大突破，投入123万元对29个无主废弃竖井进行爆破治理，关闭非法违法采选企业51家。

——社会管理得到加强和创新。基层组织建设得到全面加强，在3个社区试点推行“精街道，强社区”工作，投资610万元完成明安社区一站式服务大厅建设。大力开展“平安固阳”建设，投入300万元完善城市视频联网监控系统。深入开展隐患排查治理和安全生产大检查，安全生产形势保持稳定。在抓捕积案命案逃犯专项行动中，成功破获3起积案命案，有效震慑了违法犯罪活动，维护了社会平安稳定。加强社会工作联席会议和调解制度，继续推进重大事项社会稳定风险评估工作，全力化解信访积案，保障了群众的合法权益。全面加强食品药品安全监管，保障了人民群众饮食用药安全。

——政府自身建设不断加强。严格执行“八项规定”，控制“三公”经费支出，全面开展公务用车和办公用房清理。自觉接受县人大、县政协的监督，认真执行各项决议。加强民主法制和党风廉政建设，反腐倡廉取得明显成效。强化政务服务中心管理，实现了行政审批服务的高效便民。完善应急预案机制体制，提高了政府应对突发事件的能力。

各位代表，在看到成绩的同时，我们也清醒地认识到，固阳仍处于发展的初级阶段。贫困人口多、脱贫致富任务艰巨、小康社会建设任重道远；产业基础薄弱，结构单一，产业延伸不长，支柱产业尚未形成；税源不稳定，后续增长乏力，财政收支矛盾突出；生态保护历史欠账较多，治理私挖盗采、节能减排任务艰巨。县政府将坚定信心，直面困难，从县情出发，顺势而为，使政府工作更加符合发展的要求和老百姓的期盼。

二、2014年主要工作任务

今年，是全面落实自治区“8337”发展思路的重要之年，是全面完成“十二五”规划各项目标任务的关键之年，是加快固阳县脱贫致富进程，加快全面建设小康社会的攻坚之年。

2014年的整体发展思路是：按照党的十八届三中全会的总体部署，准确把握中央经济工作会议、城镇化工作会议和农村工作会议精神，深入贯彻落实自治区“8337”发展思路、市委十一届五次、六次全委（扩大）会议和县委十一届四次全委（扩大）会议精神，紧紧围绕“发展、民生、生态”三大主题，重点抓好“五个提质”、强力推进“两项工程”、 努力实现“一个提升”， 全力落实“建设包头及发达地区产业转型升级承接地、包头市精品卫星城、绿色生态屏障、如期实现脱贫致富”四大目标工作任务。

2014年经济社会发展的主要预期目标是：地区生产总值增长8%；固定资产投资增长15%；公共财政预算收入增长6%；社会消费品零售总额增长10%；城镇居民人均可支配收入增长9%；农民人均纯收入增长9%。确定这样的目标，是县委、县政府综合考虑改革、发展、稳定等各方面因素，经过反复研究、慎重决定的。

（一）重点抓好“五个提质”

一是多点发力促进工业园区提质。坚持把工业园区建设作为推进经济发展的主引擎，从园区规划配套、招商引资、项目建设等方面多点发力，推进园区提质扩容增效，着力打造特色鲜明的产业集群，为工业提质提供可靠支撑。

从园区规划和配套发力。严格执行园区一期20.5平方公里的控制性详规，完成二期80.5平方公里扩区方案的报批工作。集中力量完善园区基础设施，年内开工并完成管道天然气和铁路专用线的建设工作，持续推进园区道路、供水、供电、通讯等基础设施建设。重点谋划实施污水处理厂和工业物流园区等关键配套设施。积极开展园区代管行政村工作，有计划地组织实施园区内村庄搬迁。加快园区环境建设，为投资商提供一个良好、诚信、和谐的发展环境。

从招商引资发力。一是围绕产业转移开展产业招商，研究产业转移新形势、新特点，制订产业招商路线图和专项实施方案，积极主动承接市区及长三角、珠三角、京津冀的产业转移，引进科技含量高、用地集约、环保达标、符合产业政策的大项目。二是围绕产业链延伸开展以商招商，立足现有项目招商，拉长产业链条，提高产品附加值，提升产业集聚能力。三是围绕与稀土高新区共建园区开展合作招商，与高新区在项目、资金、园区建设等方面进行深度合作，通过错位发展，实现互利共赢。四是围绕领导带头开展战线招商，政府领导要亲自挂帅，结合各自分管工作开展招商活动，担负起招商主要责任人职责。

从项目建设发力。加快汇豪10万吨镁合金技改、科韵环保矿渣微晶玻璃材料等项目建设进度。尽快完善新疆特变20万千瓦风电、内蒙华电红泥井1万千瓦光伏发电等新能源项目的前期核准手续，争取4月份开工实施。帮助安徽和电普华抓紧时间办理30万吨高性能稀土铝合金电缆项目的各项审批手续，力争4月份开工建设。同时，全力争取实施2×66万千瓦级低热值煤炭发电、包钢二期240万吨球团、泰恒二期180万吨铁精粉造块200万吨铁精粉等项目。

二是转型升级带动农牧业提质。以农牧业产业园区为依托，充分发挥龙头企业资金、技术、品牌、销售渠道等优势，把我县建成绿色农畜产品加工输出基地，为农业提效提供有力支撑。

向园区发展转型。加快完成农牧业产业园区的报批工作，进一步完善规划设计，完善基础设施，健全组织机构，推进园区建设进度。重点扶持田丰、草原百盈等龙头企业做大做强，发挥其示范带动作用，引导更多的龙头企业进驻产业园区。加大农畜产品精深加工比重，实现农畜产品加工园区化，园区产业化、集聚化，延长产业链条，提高产品附加值。培育发展农畜产品和副食品加工基地，形成群众放心食品生产产业链，满足包头市及周边群众的需求。力争总投资6亿元的内蒙古西部粮食现代加工物流园区项目在固阳县实施。

向品牌发展转型。依托绿色无污染这一最大优势，切实加强原产地产品及地理标识产品的认证和保护，加快培育一批在市场上叫得响、占有率高的有机绿色、安全健康的生态农畜产品。重点打造“三主粮”燕麦、草原百盈“德吉玛”品牌羊肉、田丰马铃薯等本土品牌。认真落实自治区扶持创建著名、驰名品牌政策，引导企业加强产品质量监管，拓展市场空间，完善营销策略，增强市场竞争力。

向规模发展转型。全面完成小型农田水利重点县高效节水灌溉工程和“四个千万亩”节水灌溉项目，新增和改善高效节水灌溉面积4.3万亩，累计达到20万亩。马铃薯种植面积稳定在60万亩，逐步加大种薯繁育比例，实施脱毒种薯扩繁6万亩。继续加大土地流转力度，全面扶持各类农村合作组织。全年肉羊存栏稳定在90万只，继续推动全县羊品种的改良，提高养殖效益。不断扩大燕麦、小杂粮、中草药等特色作物的种植规模，达到20万亩。支持和鼓励发展野猪、土鸡等特色养殖，达到190万头（只）。

三是挖潜盘活拉动服务业提质。按照“发展提速、比重提高、竞争力提升”的总体要求，盘活现有商贸物流、文化旅游等资源，为三产扩量提供有效支撑。

盘活现有商业资源。认真落实《固阳县关于加快第三产业发展的扶持政策》，在建设用地、财税及金融信贷等方面采取优惠、以奖代补等形式给予支持。推动怀朔商业步行街、佳雨大酒店、鸿云大酒店、城市广场、阳光锦城商业街等商业项目启动运营。特别是要加大对集商贸、餐饮、休闲为一体的广众国际中心等大型商业综合体的政策扶持力度，支持其尽快投入运营，满足群众消费需求，提升城市品味。加快推进农村服务业发展，保证鲜活农产品运输和群众生活必需品供应。

盘活文化旅游资源。促进文化旅游融合发展，高起点、高标准编制《固阳县文化旅游发展规划》，建设旅游休闲度假基地。启动秦长城文化产业园区建设，谋划具有地方特色的文化休闲项目，推动园区常态化运营。支持加快春坤山、秦长城旅游景区开发建设进度。完善天盛城段秦长城、马鞍山、大仙山等旅游景区的基础设施，做好怀朔镇古城遗址和阿塔山水库远景规划，为招商引资和景区开发奠定基础。

挖掘物流资源潜力。按照“服务带动、物流先行”的战略，推进物流业市场化、专业化和社会化。规划建设三个物流基地，以两条一级公路、两条省道和铁路专用线为依托，围绕工业园区，建立工业物流园区，围绕农牧业产业园区，建立仓储物流园区，围绕为包头市及周边地区提供放心副食品，建立粮食现代加工物流园区。

四是拓展改造助推城镇建设提质。提升城镇品位，体现城镇特色，提升承载功能，建设包头市精品卫星城和山北地区绿色生态屏障。

拓展城区发展空间。高起点、高标准修编城区控制性详规，启动农牧业产业园区、文化产业园区、仓储物流园区规划编制，形成旧城区、新城区、产业集聚区“三位一体”的县城发展新格局。树立规划权威性，严厉打击未批先建、私搭乱建等违法违设行为，确保规划区内违法建筑“存量递减，增量为零”。 强化城市用地经营理念，用好用活土地资源，对建设用地提前储备、统一管理，依据规划适时集中连片开发，实现土地增值的最大化，提高城市改造建设的质量。

改造城市落后环节。全年力争完成投资11.2亿元，重点实施24项城建项目。下大力搞好路网建设，实施城区8条15公里道路的改扩建工程，完成98条10万平方米的小街巷治理改造工程，贯通新城区“两横两纵”道路，逐步推进新旧城区道路两侧绿化、美化、亮化。下决心抓好城市棚户区改造，在全面完成繁荣路等棚改项目的基础上，启动中市街、南关居委等3片区240户棚户区改造项目，实施好阳光锦城三期等商住项目，完成新汽车站建设搬迁工作。高品位、高标准打造梅令公园、木兰公园，规划建设一批体现地方特色的景观、景点和街区步道。规划建设垃圾转运站，加大城中村、城边村环境卫生整治力度，抓好城市形象治理工程。全面整顿治理长途客运、公交、出租车的运营秩序，塑造良好的城市窗口形象。建立完善自治区文明县城创建工作机制，夯实文明县城创建基础。

统筹推进城乡建设。按照“收缩、转移、集中”的原则，组织实施常住人口30人以下自然村整村搬迁移民工程，继续抓好哈业忽洞、巨和城、学田会等新农村示范点续建工程。加大危房改造项目争取力度，全年改造危房2300户。投入1.2亿元高标准规划建设金山镇召地新村。投入3000多万元对金山镇沿包固一级公路两侧的三分渠等5个村实施村容村貌整治工程。持续推进基础设施建设，力争开工建设固阳至武川一级公路，重点推进省道311固阳至红泥井公路，怀朔镇至兴顺西镇、怀朔镇至下湿壕镇公路以及5条23公里通村公路的建设工作。

五是多措并举力推生态环境提质。坚持不懈地抓好生态环境治理，实现呼得上新鲜空气、看得见满眼绿色，构建包头市山北地区绿色生态屏障。

加大生态环境建设力度。完成京津风沙源治理等国家林业重点工程8万亩。投入3.3亿元，完成新城区、公路通道、园区及重要节点绿化造林面积1.7万亩，突出抓好包固一级公路27公里1800亩绿化任务。全力争取国家退耕还林项目，年内力争完成20万亩。

加大生态环境保护力度。严格落实项目环境保护预审，严把建设项目环境准入关。加大环境大气和饮用水源地监测力度，确保饮用水源地水质达标率达到100%，二级以上良好天气数达到320天。加大城市小锅炉清理整治力度，逐步进行拆除。开展违法排污企业清理整治专项行动，对污染破坏环境的行为做到“零容忍”。推动一批重点企业完善环保设施和环保手续，实现依法达标排放，严守环境保护红线。依法开展清理整治非法侵占林地，违法勘查、开采矿产资源，非法采砂、私挖盗采等违法行为，关停关闭一批严重破坏生态环境的非法小选厂、小冶炼，做到“三不留，一毁闭”，实现破坏生态环境违法行为 “存量递减、增量为零”。

（二）强力推进“两项工程”

一是强力推进扶贫攻坚工程。把扶贫攻坚作为全县的重大政治任务来动员、部署、落实，按照扶贫规划的总体要求，严格落实各项扶贫措施，积极争取扶贫资金，不断扩大扶贫工作受惠面，贫困人口人均纯收入达到4600元以上。

深入推进产业扶贫。围绕主导产业推进产业扶贫，安排财政专项资金对贫困户和带动贫困户脱贫增收的龙头企业、农牧业合作社进行贴息，引导贫困农民调整种养结构，提高种养殖收入。围绕龙头企业推进产业扶贫，充分发挥田丰、草原百盈、三主粮等种养业龙头企业的示范带动作用，引导贫困农民自主选择马铃薯、燕麦、黄芪和肉羊、土鸡等种养殖项目，促进贫困农民稳定增收。同时，探索建立龙头企业与贫困农户利益共享机制，支持贫困农户以土地入股的方式参与龙头企业发展，实现风险共担、利益共享。

继续深化政策扶贫。充分用足、用好各类政策性保障功能，保障无劳动能力、难以脱贫人群的基本生活需求。采取政府补贴、社会资助、农民自筹等形式，适当调整缴费标准，逐年提高养老保险参保率和养老保险金待遇。进一步扩大农村低保覆盖面，65周岁以上农村在籍符合条件的贫困老年人全部纳入农村低保，发挥其扶贫的“兜底”功能。

深入实施金融扶贫。全力推进金融扶贫富民工程，全年投放贴息贷款不低于1亿元。通过协调农业银行等金融机构发放贷款，重点支持有生产经营能力的农户、农民专业合作组织、扶贫龙头企业，重点扶持肉、薯、药材等十大扶贫产业及能够安置大量贫困农民就业的其他劳动密集型产业，放大贷款规模，做大做强扶贫特色优势产业，加快贫困农民增收致富步伐。

全面实施定点扶贫。继续紧紧抓住自治区、包头市领导联系帮扶贫困地区和全市集中社会力量重点帮扶固阳的历史机遇，积极争取各类帮扶项目资金。整合一切涉农资金，重点用于农村互助幸福院、召地新村、农牧业产业园区和村容村貌整治等建设。全面开展规划、项目、干部“三到三定”帮扶工作，选派干部脱岗专职进村开展扶贫，确保扶贫工作成效。

二是扎实推进民生保障工程。按照“学有所教、劳有所得、老有所养、病有所医、住有所居”的总体要求，积极发展各项民生事业，使改革和发展成果实实在在惠及到固阳老百姓，为建设“幸福固阳”夯实基础。

扎实推进民生保障。实施创业就业工程，鼓励各类企业优先录用贫困农民工和失地农民工，引导贫困人口就近就地转移就业，全年城镇新增就业1500人。严格执行《固阳县治理拖欠农民工工资问题实施办法（试行）》，坚决杜绝拖欠农民工工资行为。完善城乡低保等各项救助制度，实现应保尽保。投入2000万元完成首批7处互助幸福院试点建设工作，解决农村老人特别是留守贫困老人的老有所居和老有所养问题。实施19处农村安全饮水工程，保障城乡居民用水安全。

扎实推进民生事业。完成县医院综合楼标准化建设，新建金山镇中心卫生院等5所镇卫生院和15所标准化村卫生室。进一步扩大农村合作医疗覆盖面，参合率达到99.4%。扎实推进医改各项措施，为群众提供方便价廉的公共卫生和基本医疗服务。继续为65岁以上老人实行免费体检，实现全覆盖。继续稳定低生育水平，促进人口均衡发展。完成县文化艺术中心大楼建设并面向社会开放，建成5个社区文化站和18个村文化室。继续推行文化精品工程，文明节俭组织好群众性文化活动。不断加大教育投入，稳定教师队伍，提高教育教学水平，重点实施“1+1”爱心救助等扶贫助学工程，实现贫困生救助全覆盖。

扎实推进社会管理创新。继续推行重大工程项目和重大决策的社会稳定风险评估机制，积极开展信访积案化解和源头治理工作，保障人民群众正当利益。逐步推开“精街道，强社区”工作，切实发挥好社区居委管理服务作用。认真落实企业安全生产责任制，切实加强饮用水源、食品、药品质量安全监管，认真做好重大疫病防控工作，确保安全生产和人民群众饮食用药安全。加强社会治安综合治理，完成城市视频监控系统建设，提高立体防控能力。

（三）完善政府服务，提升发展软实力

按照“为民、务实、清廉”的总要求，增强自我约束，提高管理水平，坚持依法行政，建设法治政府。

从自身建设提升软实力。扎实开展党的群众路线教育实践活动，着力解决人民群众反映强烈的突出问题。全面推进信息化建设，实现高效办公，节约办公。完善全县重大事项、重点工程通过政府门户网站公示制度，接受群众查询、监督，维护群众基本权利。增强部门协作，完善联动机制，构建“履职有力、联动高效”的部门服务新机制。坚持解放思想，建立勇于担当、效率优先、立说立行、立说立改的工作机制，树立敬畏群众、敬畏权力、敬畏组织的工作态度，营造讲实话、办实事、求实效的工作氛围。

从决策管理提升软实力。充分发挥财税、矿经等部门的职能作用，加强征管，堵塞漏洞，最大力度增加可用财力，实现保民生、保重点、保运转的总要求。严格执行项目资金争取奖惩制度，加大部门资金争取力度。加快融资平台搭建，盘活现有资产，有效缓解财政压力。进一步压缩“三公” 支出，厉行节约、杜绝浪费。

从改革创新提升软实力。以增强县域综合实力、推进社会公平正义、提升人民幸福感和满意度作为政府改革创新的主要目的。全力推动行政审批改革，凡上级明令取消的审批事项立即取消，凡下放我县的审批事项能取消的尽量取消，能简化的尽可能简化。建设乡镇便民服务中心和村级便民服务点，逐步形成“三级联动、畅通高效”的公共服务体系。围绕保障和改善民生，深化社会体制改革。完善就业、养老、医疗等社会保障制度，加强监管，确保公开透明。积极推行户籍制度改革，提高城镇化率。

各位代表，新的征程已经开启，新的目标催人奋进。让我们在市委、市政府和县委的坚强领导下，凝心聚力，攻坚克难，不负人民重托，不误发展机遇，为早日实现建设包头及发达地区产业转型升级承接地、包头市精品卫星城、绿色生态屏障、如期实现脱贫致富“四大目标”而努力奋斗！
